# Stable isotope‐based trophic structure of pelagic fish and jellyfish across natural and anthropogenic landscape gradients in a fjord estuary

**DOI:** 10.1002/ece3.2450

**Published:** 2016-10-18

**Authors:** Sean M. Naman, Correigh M. Greene, Casimir A. Rice, Joshua Chamberlin, Letitia Conway‐Cranos, Jeffery R. Cordell, Jason E. Hall, Linda D. Rhodes

**Affiliations:** ^1^ Department of ZoologyUniversity of British ColumbiaVancouverBCCanada; ^2^ NOAA FisheriesNorthwest Fisheries Science CenterSeattleWAUSA; ^3^ NOAA FisheriesMukilteo Research StationMukilteoWAUSA; ^4^ School of Aquatic and Fishery SciencesUniversity of WashingtonSeattleWAUSA; ^5^ Present address: Washington Department of Fish and WildlifeHabitat ProgramOlympiaWAUSA

**Keywords:** fish, food webs, jellyfish, Puget Sound, stable isotopes, trophic structure

## Abstract

Identifying causes of structural ecosystem shifts often requires understanding trophic structure, an important determinant of energy flow in ecological communities. In coastal pelagic ecosystems worldwide, increasing jellyfish (Cnidaria and Ctenophora) at the expense of small fish has been linked to anthropogenic alteration of basal trophic pathways. However, this hypothesis remains untested in part because baseline description of fish–jellyfish trophic dynamics, and the environmental features that influence them are lacking. Using stable isotopes of carbon (δ^13^C) and nitrogen (δ^15^N), we examined spatiotemporal patterns of fish and jellyfish trophic structure in greater Puget Sound, an urbanizing fjord estuary in the NW United States. We quantified niche positions of constituent species, niche widths and trophic overlap between fish and jellyfish assemblages, and several community‐level trophic diversity metrics (resource diversity, trophic length, and niche widths) of fish and jellyfish combined. We then related assemblage‐ and community‐level measures to landscape gradients of terrestrial–marine connectivity and anthropogenic influence in adjacent catchments. Relative niche positions among species varied considerably and displayed no clear pattern except that fish generally had higher δ^15^N and lower δ^13^C relative to jellyfish, which resulted in low assemblage‐level trophic overlap. Fish assemblages had larger niche widths than jellyfish in most cases and, along with whole community trophic diversity, exhibited contrasting seasonal patterns across oceanographic basins, which was positively correlated to landscape variation in terrestrial connectivity. In contrast, jellyfish niche widths were unrelated to terrestrial connectivity, but weakly negatively correlated to urban land use in adjacent catchments. Our results indicate that fish–jellyfish trophic structure is highly heterogeneous and that disparate processes may underlie the trophic ecology of these taxa; consequently, they may respond divergently to environmental change. In addition, spatiotemporal variation in ecosystem connectivity, in this case through freshwater influence, may influence trophic structure across heterogeneous landscapes.

## 1 Introduction

Trophic structure is a key determinant of energy flow within ecological communities (Hairston & Hairston, 1993). Because altered energetic processes often underlie shifts in aggregate community properties including composition and abundance, characterizing trophic structure is critical to understand both natural and anthropogenic changes to ecosystems (Rooney, McCann, & Moore, 2008). For instance, reduced trophic complexity following environmental degradation may indicate incipient impacts to abundance or species diversity (Layman, Quattrochi, Peyer, & Allgeier, 2007; Tewfik, Rasmussen, & McCann, 2005). This has been increasingly recognized in an applied context, and a growing number of studies have taken more holistic, trophic‐based approaches to tackle complex management and conservation issues (Gray et al., 2014).

An issue in pressing need of attention in many coastal and estuarine ecosystems involves compositional shifts in mid‐trophic level pelagic communities, where fish are declining and jellyfish (Cnidarians and Ctenophores) are increasing (Flynn et al., 2012; Greene, Kuehne, Rice, Fresh, & Penttila, 2015; Purcell, Uye, & Lo, 2007; Richardson, Bakun, Hays, & Gibbons, 2009). Given that small pelagic fish are important regulators of energy flow (Cury et al., 2000; Robinson et al. 2014; Ruzicka et al. 2012) and jellyfish have relatively few predators (Richardson et al., 2009), these compositional shifts may indicate reduced capacity to support higher trophic levels in coastal ecosystems.

Alteration of underlying trophic structure has been implicated as a potential mechanism for pelagic compositional shifts. Specifically, some studies have posited that declining water quality associated with eutrophication modifies conditions of primary production, leading to altered trophic pathways that favor jellyfish over fish (“the bifurcated food web hypothesis”; Parsons & Lalli, 2002). However, because many aspects of coastal pelagic systems are often poorly characterized, this idea remains untested empirically, as do other hypotheses of compositional shifts including natural variability (Condon et al., 2012; Nagata, Moreira, Pimentel, & Morandini, 2015), substrate hardening, overfishing, and climate change (Richardson et al., 2009).

Characterizing fish–jellyfish trophic dynamics and the environmental features that may influence them is a critical starting point to explore hypotheses of what drives compositional shifts. For instance, if anthropogenic alteration of basal trophic pathways is an important mechanism, then fish–jellyfish trophic structure should be related to landscape gradients of human influence. Specifically, the bifurcated food web hypothesis predicts shorter food chain length, reduced trophic diversity, and lower niche overlap between fish and jellyfish as jellyfish consume lower trophic‐level prey that is unavailable to fish in more eutrophic areas (Parsons & Lalli, 2002; Purcell, 2012). Similarly, local oceanographic conditions including freshwater and terrestrial influence may also be important for fish–jellyfish trophic dynamics insofar as it determines diversity, productivity (Kimmerer, 2002), and bacterial processing rates (Bell, Bluhm, & Iken, 2012) in lower trophic levels. Consequently, we may expect greater trophic diversity (e.g., longer food chains and wider niche breadths) and lower trophic overlap in more terrestrially influenced areas (Abrantes, Barnett, Marwick, & Bouillon, 2013).

Stable isotope analysis (SIA) of carbon and nitrogen provides a powerful tool for the empirical measurement of trophic structure by elucidating the relative trophic positions of consumers within a community and the contributions of distinct basal resources (Peterson & Fry, 1987). In addition, SIA methods have been extended to measure trophic structure of whole assemblages or communities (Jackson, Inger, Parnell, & Bearhop, 2011; Layman, Arrington, Montana, & Post, 2007), thus permitting empirical measurement of emergent responses to natural and anthropogenic sources of environmental variation (Layman et al., 2012; Mancinelli & Vizzini, 2015); for instance, across gradients of saltwater intrusion in estuaries (Abrantes, Barnett, & Bouillon, 2014).

While several studies have used SIA and community‐level metrics to describe fish and jellyfish trophic structure (e.g., Brodeur, Suchman, Reese, Miller, & Daly, 2008; Brodeur, Sugisaki, & Hunt, 2002; Nagata et al., 2015), they have not explicitly incorporated environmental variation into their designs. Thus, the controls on fish–jellyfish trophic structure remain poorly defined. We address this gap using a large‐scale field study in Puget Sound, WA, USA, an urbanizing fjord estuary fed by a complex network of rivers (Strickland, 1983). Strong evidence for human‐induced ecosystem shifts in Puget Sound (Brandenberger, Louchouarn, & Crecelius, 2011; Greene et al., 2015) led to a comprehensive study of nearshore pelagic food webs across the region (Greene et al., 2012, 2015). As a component of this work, we measured carbon and nitrogen stable isotopes of fish and jellyfish along with environmental and land‐use parameters across six oceanographic basins in Puget Sound in spring, summer, and fall. Our specific objectives were to: (1) broadly characterize trophic relationships among common pelagic fish and jellyfish species; (2) describe spatiotemporal variation in the trophic structure of fish and jellyfish assemblages and their potential overlap, and whole mid‐trophic level communities; and (3) relate these assemblage‐ and community‐level measures to landscape gradients of environmental conditions, including human influence in adjacent catchments.

## 2 Materials and Methods

### 2.1 Study site

**Figure 1:**
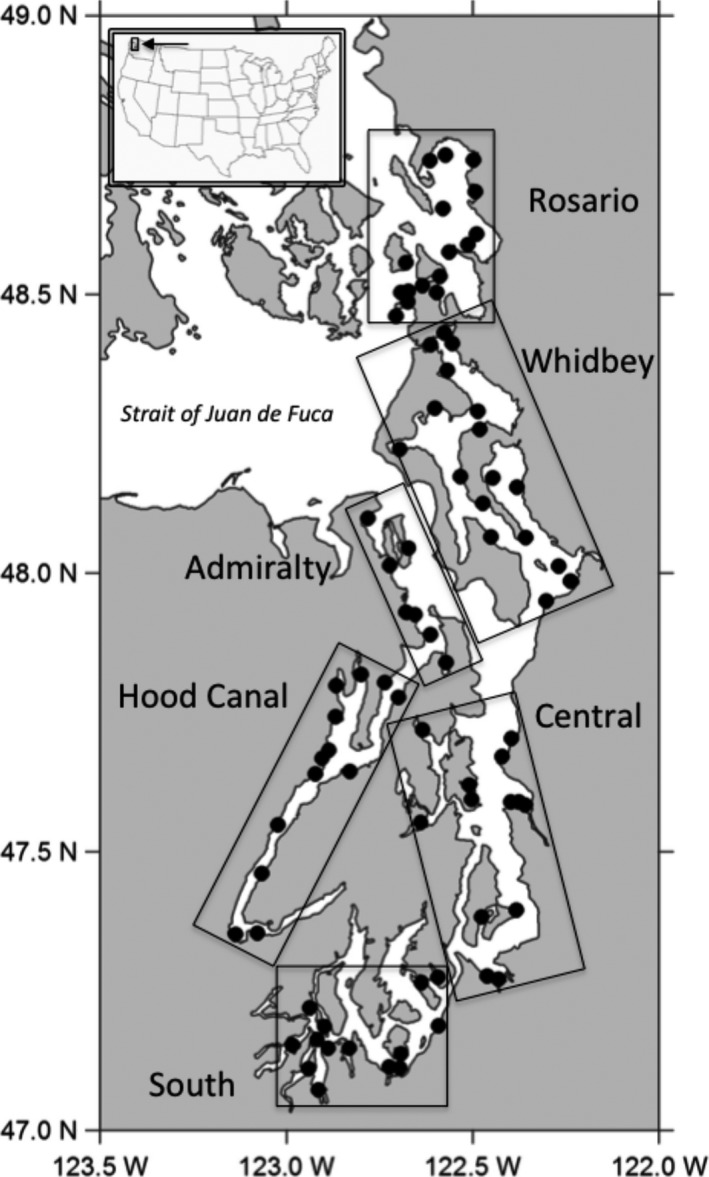
Map of sampling locations across the six oceanographic basins in Puget Sound. Each site (black circles) was sampled monthly from April to October 2011

Puget Sound is an elongate glacial fjord comprising the US portion of the Salish Sea, extending 280 km in length and encompassing over 4,000 km of shoreline (Fig. 1). While it is a marine‐dominated system, local conditions are influenced by seasonally variable inputs of freshwater from 15 major rivers (Banas et al., 2014). The six distinct oceanographic basins in Puget Sound are separated by sills and other landforms (Burns, 1985), and each has a unique set of abiotic conditions (e.g., freshwater input, residence time, and tidal influence; Moore et al., 2008). The degree of anthropogenic influence also varies among basins. Central basin and parts of South Sound are highly urbanized and have experienced significant alteration through shoreline modification (Simenstad et al., 2011; Toft, Cordell, Simenstad, & Stamatiou, 2007) as well as runoff (Newton, Anderson, van Voorhis, Maloy, & Siegel, 2002; Oyafuso et al., 2015). Other basins (Whidbey and Rosario) have been less developed but have significant agricultural areas in their catchments. In contrast, Hood Canal and Admiralty Inlet are primarily forested.

### 2.2 Field sampling

**Figure 2:**
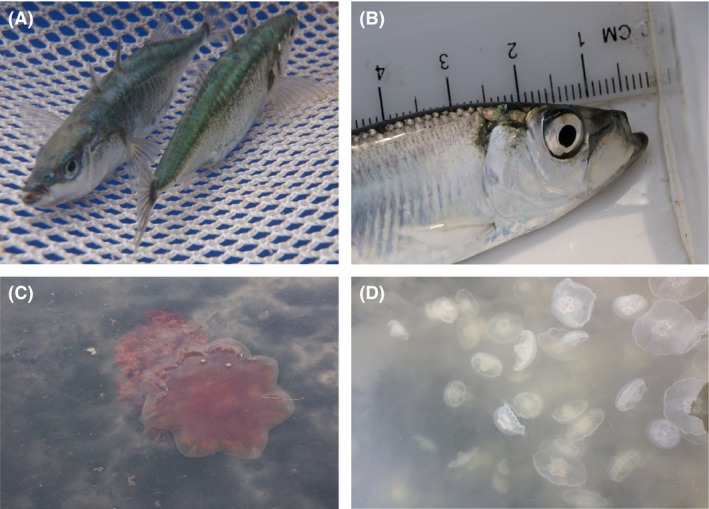
Several fish and jellyfish species collected during our study. (A) Three‐spined stickleback *Gasterosteus aculeatus* [photograph: Sean Naman], (B) Pacific herring *Clupea pallasii* [photograph: Joel Rogers], (C) Lion's mane *Cyanea capillata*, and (D) moon jellyfish *Aurelia* spp. [photographs: Correigh Greene]

As part of a larger study on Puget Sound's pelagic food web (see Greene et al., 2015), we sampled 79 sites across the six oceanographic basins in Puget Sound (Fig. 1) monthly from April to October 2011. Site selection incorporated the maximum variation of shoreline and catchment land use across the region (see Greene et al., 2012). At each site, fish and jellyfish were collected using a Kodiak surface trawl or “townet” (described in Greene et al., 2015). Up to ten individual fish and jellyfish of each species at each site were sacrificed and frozen at −20°C for stable isotope analysis. While the species composition and relative abundance varied spatially and temporally across the study, several species were consistently present including fish species: juvenile Chinook salmon *Oncorhynchus tshawytscha* (45% frequency of occurrence), three‐spined stickleback *Gasterosteus aculeatus* (42%; Fig 2A), juvenile chum salmon *O. keta* (32%), Pacific herring *Clupea pallasii* (29%; Fig 2B), and surf smelt *Hypomesus pretiosus* (22%); and jellyfish species: sea gooseberry *Pleurobrachia bachei* (58%), water jelly *Aequorea* spp. (57%), cross jelly *Mitrocoma* spp. (35%), and lion's mane *Cyanea capillata* (24%; Fig 2C). Other more patchily distributed species were collected when available including: juvenile coho salmon *O. kisutch* (11%), bay pipefish *Syngnathus leptorhynchus* (7%), Pacific sandlance *Ammodytes hexapterus* (6%), northern anchovy *Engraulis mordax* (5%), moon jelly *Aurelia* spp. (13%; Fig 2D), and fried egg jelly *Phacellophora camtschatica* (7%). Fork length of fish and bell diameter of jellyfish were recorded during collection. An effort was made to sample across a consistent range of sizes; however, body size of many species varied considerably throughout the study (C. Greene, Unpublished). Implications of this variation are discussed in Appendix S2.

### 2.3 Laboratory methodology

In total, 1,078 fish and 728 jellyfish samples were analyzed for ^15^N and ^13^C (see Table S1). For fish, dorsal muscle plugs were extracted from each individual, freeze‐dried for 24 h, and then ground into a homogenous powder. To obtain sufficient material for analysis, whole body samples of individual jellyfish of each species were pooled for a given site. An effort was made to only combine samples of jellyfish of similar size. For cases of large individuals, we combined bell and digestive tract tissue to ensure consistency with pooled samples. Jellyfish samples were dried at 60°C for 24 h and then ground into fine powder. All samples were then weighed into specified amounts (0.5–0.8 mg for fish, 0.5–10 mg for jellyfish) and then analyzed for stable isotope composition of ^15^N and ^13^C using a Costech Elemental analyzer and a Thermo‐Finnegan continuous flow mass spectrometer at the Northwest Fisheries Science Center and Washington State University Stable Isotope Core Lab. Isotope values are expressed in the δ notation:

$$\delta \left(‰\right)=[\left(R_{\mathrm{sample}}-R_{\mathrm{standard}}\right)/R_{\mathrm{standard}}]\times 1000$$

where *R* is the ratio of heavy to light isotope in both a sample and a standard. The standard for N was atmospheric nitrogen, and the standard for C was Vienna Pee Dee Belemnite. Standard reference material analyzed at both facilities was within ±0.01‰, indicating the results were comparable. Duplicate samples (1% of total) were run for quality assurance and were within ±0.1‰ for both isotopes. To minimize potential bias caused by variable lipid content, δ^13^C values were normalized using equations from Post et al. (2007) for fish and D'Ambra, Carmichael, and Graham (2014) for jellyfish in cases (*n *=* *344) where the C‐to‐N ratio exceeded 3.5 (Post et al., 2007).

### 2.4 Data analysis

For all analyses, site‐level data were pooled spatially for each basin and temporally into three seasons: spring (April, May, June), summer (July, August), and fall (September, October), which corresponded to periods of similar abiotic conditions (Greene et al., 2012). While low sample sizes precluded formal analysis at the species level, qualitative inference into the trophic relationships among pelagic species in each basin and season were made through graphical examination of δ^13^C and δ^15^N values (means and standard deviations). To standardize for trophic baseline variability, the relative trophic level (TL) was estimated using the formula from Post (2002):

$$\text{TL}=\lambda +(\delta {}^{15}N_{\mathrm{consumer}}-\delta {}^{15}N_{\mathrm{base}})/\Delta$$

where λ is the trophic level of δ^15^N_base_ and Δ is the trophic fractionation, which we assumed to be 3.4‰ (Post, 2002). For δ^15^N_base_, we used Pacific oyster *Crassostrea gigas* from studies in specific basins of Puget Sound (Conway‐Cranos et al., 2015; Ruesink, Trimble, Berry, Sprenger, & Dethier, 2014) and assumed a λ value of 2. Previous studies have shown that temporal variability in primary consumer isotopic composition in Puget Sound is small relative to spatial variability (Howe & Simenstad, 2015; Ruesink et al., 2014); thus, we assumed using data from earlier years introduced minimal biases to our results. Further investigation of trophic baseline variation is presented in Appendix S1.

At the assemblage level, we determined whether fish and jellyfish occupied distinct isotopic niches using a permutational multivariate analysis of variance (PERMANOVA; Anderson, 2001). Stable isotope data were normalized by subtracting means and dividing by SD to place on comparable measurement scales and to homogenize variances between groups. Then, a resemblance matrix was computed using Euclidean distances (Dethier, Sosik, Galloway, Duggins, & Simenstad, 2013) and a PERMANOVA model was fit to this distance matrix using the *adonis* function in the vegan package in R (Oksanen et al., 2013; R Core Team 2013). *adonis* is similar to traditional ANOVA and returns a pseudo *F*‐statistic and *p*‐Value based on 999 permutations of the data (Dixon, 2003). In our case, the model tested the null hypothesis that fish and jellyfish do not occupy distinct isotopic niches (i.e., are “fully mixed” in CN space). Basin and season were used as strata in this analysis (Anderson, 2001).

### 2.5 Community and assemblage trophic structure metrics

For community‐level analyses, all fish and jellyfish were pooled for each basin–season combination. We then quantified trophic structure using a series of metrics, originally proposed by Layman, Arrington et al. (2007), based on the spread of the δ^13^C and δ^15^N values of individual consumers in CN space. Each metric gives distinct insights into the trophic structure. The *nitrogen range* (NR) and *carbon range* (CR) indicate the distance between individuals with the highest and lowest δ ^15^N and δ^13^C value, respectively. NR is a measure of the trophic length, and CR indicates the diversity of basal resources. The *mean distance to the centroid* (CD) is calculated as the mean Euclidian distance of each individual to the centroid of that population or community and is a measure of the average trophic diversity. To estimate niche widths, that is, the total trophic diversity in a given assemblage or community, we followed the approach advocated by Jackson et al. (2011) and use *standard ellipse areas* corrected for small sample sizes (SEA_c_). Standard ellipses are calculated from the variance and covariance of δ^13^C and δ^15^N and represent the core isotopic niche that is invariant to sample size differences among groups. All metrics were calculated using the R (R Core Team V. 3.2.3) statistical package SIBER (Jackson et al., 2011). To quantify uncertainty, all metrics were resampled (*n *=* *10,000 iterations) and 95% credible intervals (CIs) were determined following the Bayesian procedure outlined in Jackson et al. (2011).

To compare fish and jellyfish assemblages, all species were classified as either *fish* or *jellyfish* within each basin and season*,* and then, niche widths for each group were calculated separately. Ellipses were resampled as described above, and the relative difference in SEA_c_ between fish and jellyfish was calculated each iteration. We interpreted differences in niche widths by examining whether the 95% credible intervals of the relative difference overlapped zero; if they did not, this would indicate that the SEA_c_ of one group is larger than the other more than 95% of the time. To assess the potential trophic overlap between fish and jellyfish, we computed an index based on the area of overlap between the two ellipses in CN space standardized to a ratio that ranged between 0 (no overlap) and 1 (complete overlap). For each iteration, overlap was calculated as the area of ellipse 1 overlapping with the ellipse 2, standardized by the area of ellipse 1. We define *fish–jellyfish* as the proportion of fish ellipses overlapping with jellyfish ellipses and *jellyfish–fish* as the proportion of jellyfish ellipses overlapping with fish ellipses. Basin–season differences were assessed by comparing 95% credible intervals. We considered an overlap ratio >0.6 to be biologically significant and indicative of the potential for competition (Guzzo, Haffner, Legler, Rush, & Fisk, 2013). Potentially confounding effects of trophic baseline variation and variable species richness are addressed in Appendix S1.

### 2.6 Environmental determinants of trophic structure

To explore potential drivers of trophic structure across Puget Sound, we used a series of abiotic variables measured at each site during the study and landscape variables of adjacent shorelines and catchments measured using GIS (Table 1). Further details on the methodology to collect these variables can be found in Greene et al. (2012) and Oyafuso et al. (2015). Community attributes of zooplankton, collected from each site (see Table 1 and Appendix S3 for sampling methodology), were also included to give insights into the influence of lower trophic levels. Because of the large number of explanatory variables relative to the sample size (*n *=* *18) and a high degree of autocorrelation, we reduced our explanatory variable set using principal components analysis (PCA). PCA reduces predictor variables into principle components; orthogonal combinations of variables that retain the maximum variation present in the original dataset. Principal components were extracted from the correlation matrix of predictor variables, and variable loadings were calculated, which represent the contribution of each predictor to a given PC axis (Jolliffe, 2002). We interpreted a loading above 0.3 as a significant contribution by a variable to a given axis (Peres‐Neto, Jackson, & Somers, 2003).

**Table 1:** A list and brief description of the abiotic and biotic variables incorporated in the principal components analysis. Full description of the methodology to collect each metric is given in Greene et al. (2012, 2015) and Oyafuso et al. (2015)

Metric	Description
Adjacent shoreline and catchment characteristics	
% Developed	Shoreline units for each site were determined from Puget Sound Ecosystem Restoration (PSNERP) drift cell framework. Land development classes were selected based on C‐CAP 2006 land cover classes at 30‐m resolution. Basin‐level land use was determined as the average of each land‐use metric at each site. Riverine inputs were determined by summing the total discharge from all gauged freshwater inputs (data available at: http://waterdata.usgs.gov/wa/nwis/current/?type=flow) to a given basin and season.
% Agriculture	
% Impervious	
Catchment area (km^2^)	
Shoreline length (km)	
Riverine input (m^3^)	
Physical and chemical parameters	
Salinity	Water column profile data (salinity, temperature and turbidity) were collected using a Sea Bird^®^ SEACAT CTD (SBE19plusV2) at 0.5 m increments. Nutrients and Chl‐*a* were analyzed from water collected from 6 m depth at each site using a 5L Niskin^®^ grab. All data were aggregated as the mean for each basin and season
Temperature (°C)	
Turbidity (NTU)	
Chl‐*a* (μg/L)	
NO_3_^−^ (μmol/L)	
Si(OH)4 (μmol/L)	
Lower trophic‐level characteristics	
Total zooplankton (no. m^−3^)	Samples were identified to the lowest possible taxonomic level then aggregated into distinct subsets. Total zooplankton describes the total zooplankton filtered; ichthyoplankton is the total larval fish and fish eggs, gelatinous zooplankton is the total Cnidaria and Ctenophora. Zooplankton was further categorized into distinct feeding groups and habitat groups based on previous literature (J. Cordell *Unpublished*). Metrics then described the relative proportion of a given feeding group relative to others. All zooplankton metrics were the site‐level average across basins and seasons.
Ichthyoplankton (no. m^−3^)	
Gelatinous zooplankton (no. m^−3^)	
Zooplankton diversity (no. taxa)	
Relative predator abundance	
Relative omnivore abundance	
Relative grazer abundance	
Relative abundance of freshwater and nearshore (FW‐NS) taxa.	

The first three PC axes explained 61% of the variation in the predictor variables and contained variable loading combinations that were interpretable (Table 2). The first axis explained 24% of the variance and was positively loaded by riverine discharge (0.35), shoreline length (0.33) and abundance of freshwater and nearshore‐associated (FW‐NS) zooplankton taxa (0.31), and negatively loaded with salinity (−0.35). We interpreted this axis as representing the degree of terrestrial–marine connectivity, where increases along this axis represent a larger influence of freshwater (higher riverine inputs, lower salinity), larger interface with the shoreline, and a more nearshore‐associated zooplankton composition. The second axis explained 21% of the variance and was positively loaded by the percent of urban development (0.43) and impervious surfaces (0.40) in adjacent catchments, and the abundance of gelatinous zooplankton (0.47). We interpreted this axis as representing the extent of urbanization. The third axis explained 18% of the variance and was positively loaded by chlorophyll‐*a* concentration (0.32) and ichthyoplankton abundance (0.38), and negatively loaded by nitrate (−0.46) and silicate (−0.34) concentrations and percent agriculture (−0.33). We interpreted the third PC axis as representing nutrient loading from agriculture. Our PCA axes thus represent three environmental gradients that may influence fish and jellyfish trophic structure across the Puget Sound: freshwater and terrestrial influence (henceforth “terrestrial connectivity”); urbanization; and agricultural influence.

**Table 2:** Variable loadings for the first three principal components with strong loadings (>0.3) are shown in bold

Metric	PC1	PC2	PC3
Catchment Area	0.29	0.22	−0.13
% Developed	0.06	**0.43**	−0.02
% Agriculture	0.25	−0.23	−**0.33**
% Impervious Surface	0.03	**0.4**	0.01
Shoreline Length	**0.33**	0.07	−0.24
Chl‐*a*	0.27	0.17	**0.32**
Salinity	−**0.35**	0.08	−0.14
Turbidity	0.27	−0.18	−0.23
Temperature	−0.25	0.06	0.11
Nitrate	−0.03	0.15	−**0.46**
Silicate	−0.03	0.15	−**0.39**
Riverine Discharge	**0.35**	−0.15	−0.16
Total Zooplankton	0.16	0.24	0.22
Predator Abundance	−0.25	−0.08	−0.13
Omnivore Abundance	0.11	−0.28	0.09
Grazer Abundance	−0.17	−0.16	0.02
Ichthyoplankton	0.09	−0.13	**0.34**
Zooplankton Diversity	0.22	−0.07	0.09
Gelatinous Zooplankton	0.01	**0.47**	0
FW‐NS Zooplankton	**0.31**	0.01	0.17

We used correlation analysis to relate these three landscape gradients to fish and jellyfish trophic structure. Correlation analysis was selected over more complex approaches (e.g., multiple regression) as we were primarily interested in exploring general associations between variables rather than specific parameter estimation and sample sizes were low. We computed Pearson correlation coefficients between each trophic structure metric (SEA_c_, CR, NR, CD, SEA_Fish_, SEA_Jellyfish_, fish–jellyfish overlap, and jellyfish–fish overlap) and the first three PC axes and calculated *p*‐Values testing the null hypothesis that each pairwise correlation was 0.

## 3 Results

### 3.1 Species‐level patterns

**Figure 3:**
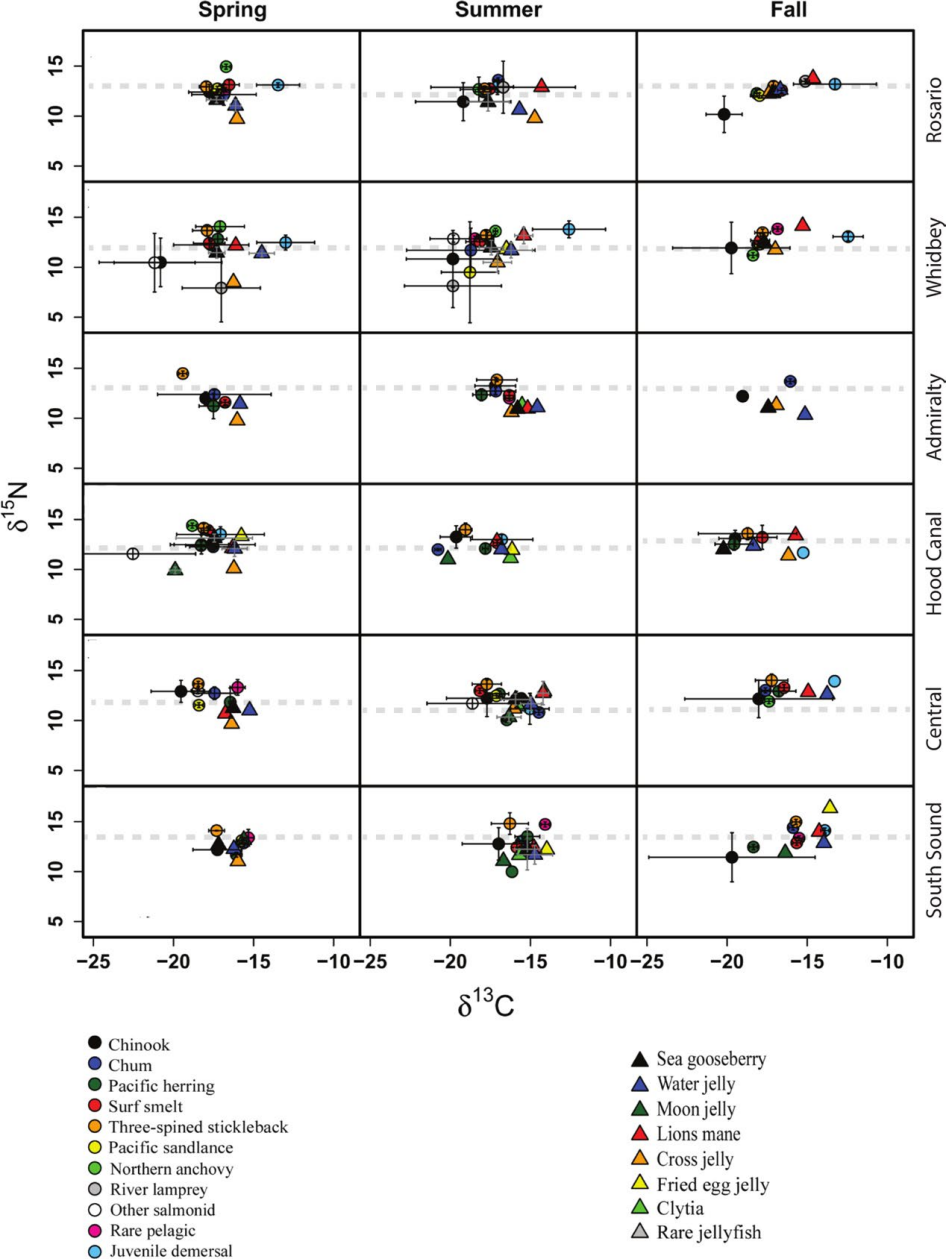
Biplots of δ^13^C and δ^15^N values (means ± SD) of the dominant pelagic fish and jellyfish species in each basin/season combinations. Basins are ordered as: (A) Rosario, (B) Whidbey, (C) Admiralty, (D) Hood Canal, (E) Central, and (F) South Sound. “Other salmonid” category includes coho, steelhead *O. mykiss*, and sockeye *O. nerka*. “Juvenile demersal” includes bay pipefish, starry flounder, and Pacific sandfish *Trichodon trichodon*. “Rare pelagic” includes tubesnout *Aulorhynchus flavidus*, squid *Loligo* spp., and Pacific tomcod *Microgadus proximus*. Dashes represent δ^15^N values corresponding to trophic level 3

Our analysis revealed context‐dependent isotopic niche positions and relationships among 25 common pelagic fish and jellyfish species across Puget Sound. Both relative and absolute niche positions among species, and pairwise overlap based on error bars appeared to vary unpredictably across basins and seasons (Fig. 3). Despite this variability, trophic differentiation was apparent between fish and jellyfish in many instances, with fish depleted in ^13^C and enriched in ^15^N relative to jellyfish. This observation was corroborated by the PERMANOVA at the assemblage level, which indicated assemblage type was a significant predictor of trophic similarity (*F*_1,1392_ = 126.1, *p *=* *.001). Notable exceptions to this pattern were fried egg and lion's mane jellyfish, which were often at higher or similar trophic levels relative to fish, and moon jellyfish, which were more depleted in ^13^C than other jellyfish species in Hood Canal and South Sound (Fig. 3). Similarly, juvenile demersal fish, including bay pipefish and starry flounder, generally occupied a higher trophic level and were more enriched in ^13^C relative to other fish and jellyfish in most basins and seasons where they occurred.

Another broad pattern was that variation in niche position among species was primarily along the δ^13^C axis. An exception to this was Whidbey Basin in spring and summer, where several species, including Pacific sandlance, river lamprey, and cross jellyfish, occupied distinct positions at lower trophic levels (Fig. 3). Within‐species variation, inferred by error bars, also tended to be higher along the δ^13^C axis. Generally, this intraspecific variation was larger in fish, especially salmonids, relative to jellyfish. Interestingly, there was little evidence for marked seasonal shifts in trophic position and weak relationships between individual body size and δ^15^N (Appendix S2). A notable exception was fried egg jellyfish, which became 4‰ more enriched in δ^15^N from summer to fall in South Sound (Fig. 3) and had the largest variation in size and the strongest relationship between bell diameter and δ^15^N (Table S4).

### 3.2 Assemblage‐ and community‐level patterns

**Figure 4:**
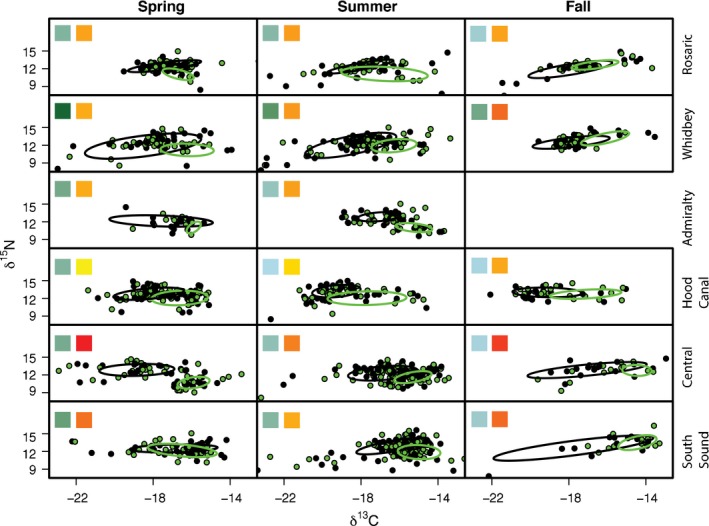
Biplots of δ^13^C and δ^15^N values showing standard ellipses drawn around assemblages of individual fish (black) and jellyfish (green) for each basin and season, ordered in the same manner as Fig. 2. Colored squares in each panel represent the relative terrestrial connectivity (blue to green) and anthropogenic influence (yellow to red) among basins. More terrestrially influenced basins are darker green, and more urbanized basins are darker red. Gradients are based on the first two PCA axes. Admiralty fall is not included due to an insufficient sample size

**Figure 5:**
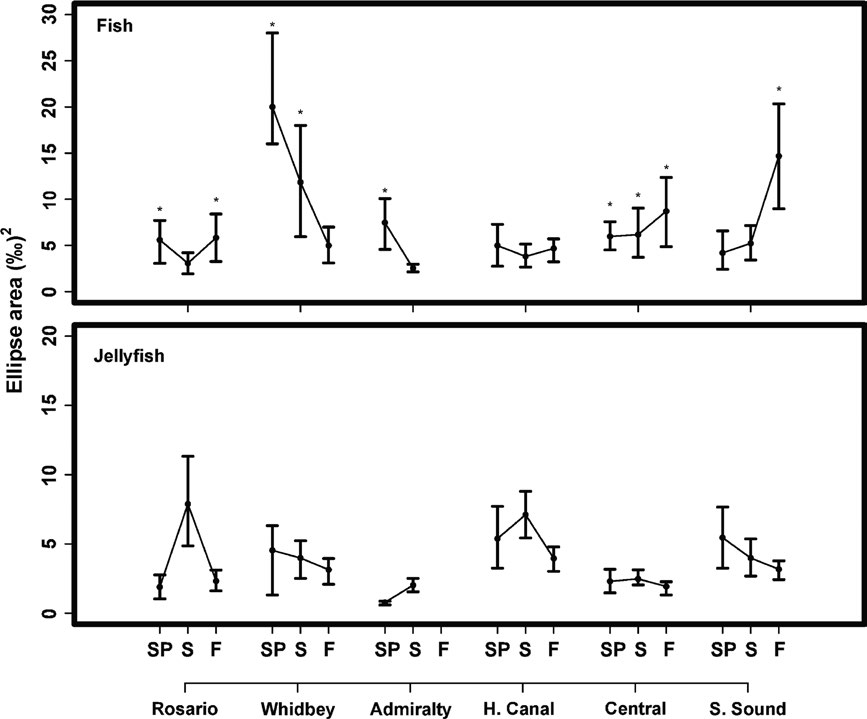
Estimates of SEA_c_ (‰^2^) for fish and jellyfish assemblages. Each point represents the mode of 10,000 iterations (±95% credible intervals). Basins are ordered north to south and seasons, spring (sp), summer (s), and fall (f), are oriented from left to right. Asterisks represent cases where SEA_c_ for a given assemblage was greater than the other more than 95% of the time

**Figure 6:**
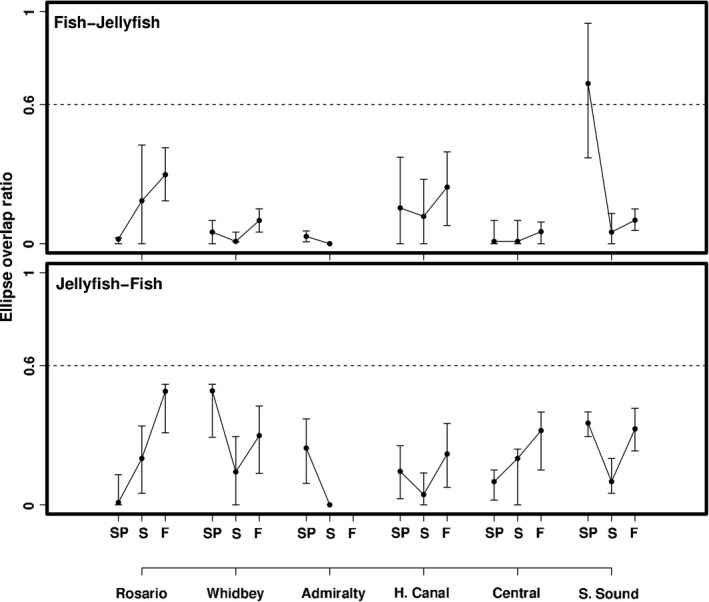
Bayesian estimates of the trophic overlap ratio between fish and jellyfish. Overlap was calculated as the area of ellipse 1 overlapping with the ellipse 2, standardized by the area of ellipse 1. *Fish–jellyfish* shows the proportion of fish ellipses overlapping with jellyfish, and *jellyfish–fish* shows the proportion of jellyfish ellipses overlapping with fish for each basin and season. Points represent the mode of 10,000 iterations (±95% credible intervals). The horizontal line at 0.6 indicates a threshold of a biologically significant overlap. Basins and seasons are configured in the same manner as Figure 5

Community‐ and assemblage‐level metrics were robust to a number of assumptions including trophic baseline variability, species richness effects, and potential habitat shifts (Appendix S1). At the assemblage level, fish and jellyfish niche widths showed distinct spatiotemporal patterns (Fig. 4). For fish, seasonal patterns of SEA_c_ varied among basins. For instance, SEA_c_ estimates in Whidbey were 2–4 times higher than other basins in spring and summer then declined into the fall (Fig. 5), while SEA_c_ in Central and South Sound increased from spring to fall. In contrast to fish, jellyfish generally had less variable niche widths among basins and seasons aside from a notable summer peak in Rosario (Fig. 5). Additionally, in most cases, 95% credible intervals of SEA_c_ differences indicated SEA_c_ for fish was larger than jellyfish. Assemblage‐level trophic overlap varied substantially (over 2 × for jellyfish and 3 × for fish) among basins and seasons (Fig. 6) but was generally low (<0.6), the exception being South Sound in spring, where fish–jellyfish overlap was 0.69 (0.38–0.95). In accordance with the species‐level results, low assemblage‐level overlap appeared to result from fish being more enriched in ^15^N and depleted in ^13^C relative to jellyfish (Fig. 4).

**Figure 7:**
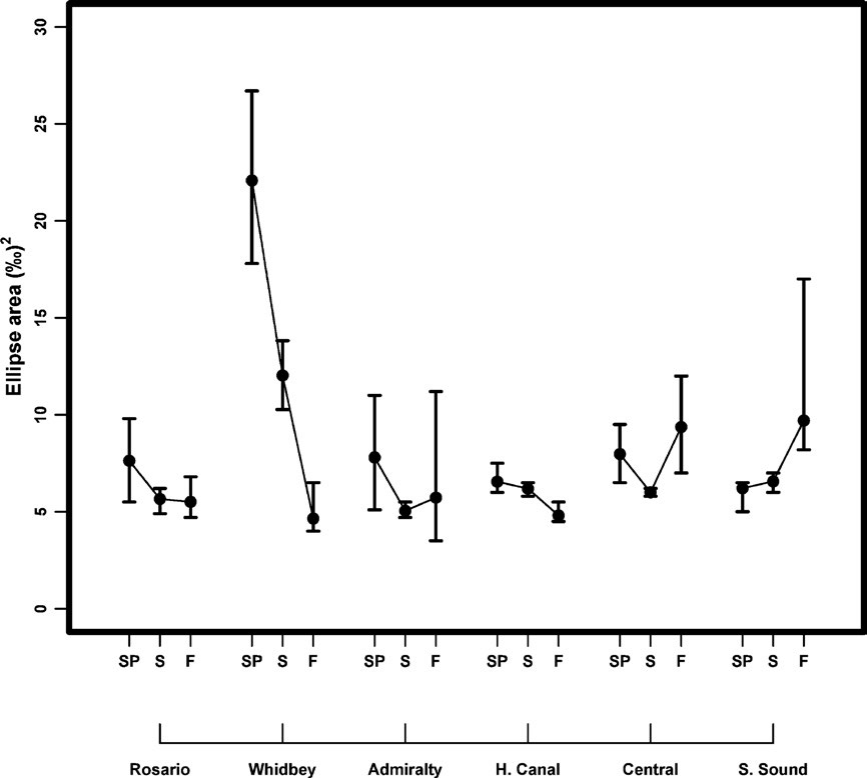
Bayesian estimates of whole community SEA_c_ (‰^2^) for each basin and season. Points are the mode of 10,000 (±95% credible intervals) iterations. Basins and seasons are configured in the same manner as Figure 5

Trophic structure metrics at the community level also exhibited contrasting seasonal patterns among basins. Community‐wide niche widths in Rosario and Whidbey were largest in the spring then contracted seasonally, while the converse occurred in Central and South Sound. Hood Canal and Admiralty generally had smaller ellipses with less distinct seasonal patterns (Fig. 7). Based on Bayesian estimates, the greatest magnitude of seasonal change occurred in Whidbey, where modal niche widths contracted over fourfold from spring to fall (Fig. 7). Niche widths in Whidbey were two to three times larger than other basins in spring (95% CIs nonoverlapping; 17.8–26.7‰^2^) and summer (10.27–13.82‰^2^). In contrast, niche widths increased 1.2 times from summer to fall in Central Basin, and 1.5 times from spring to fall in South Sound. In fall, SEA_c_ was highest in Central (6.5–12.4‰^2^) and South Sound (8.1–18.3‰^2^).

**Figure 8:**
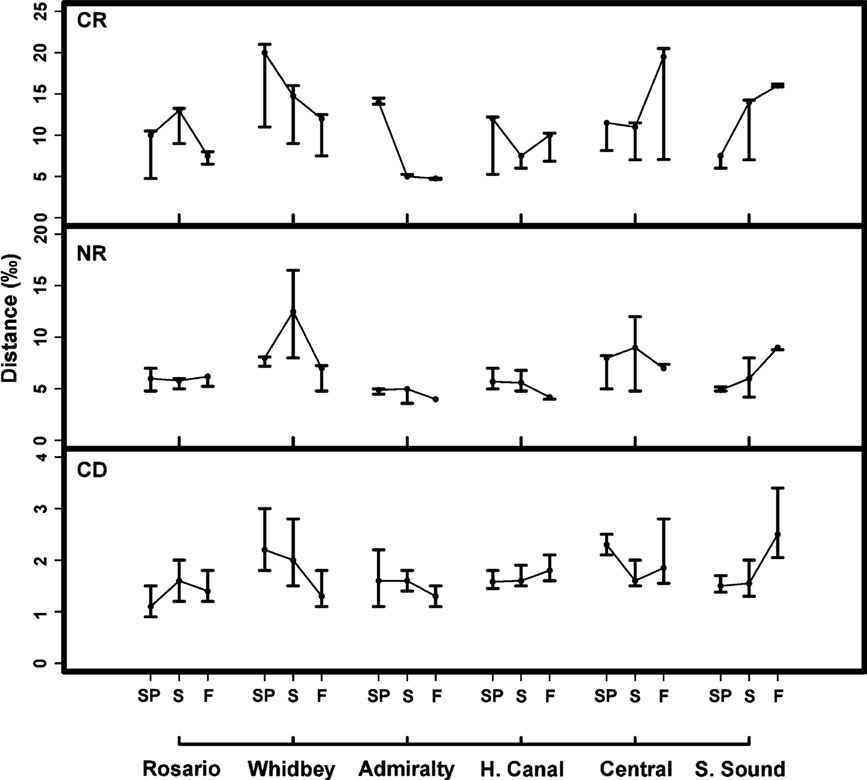
Bayesian estimates of whole community CR, the distance (‰) from the individual with the lowest to highest δ^13^C values; NR, the distance (‰) from the individual with the lowest to highest δ^15^N values; and CD, the mean distance of each individual from the centroid for each basin and season. Each point represents the mode of 10,000 iterations (±95% credible intervals). Basins and seasons are configured in the same manner as Figure 5

Spatial and seasonal trends were also evident in community‐wide basal resource diversity, trophic length, and diversity. CR was the most variable among basins and seasons, with patterns in general concordance with community niche widths (Fig. 8). In contrast, NR was less variable, but showed notable seasonal trends in Whidbey, which peaked in summer (7.5–15.2‰), and South Sound, which increased 1.3 times from spring to fall (Fig. 8). Variability in CD was also low but seasonal patterns paralleled those of SEA_c_ and CR. There was a 1.8‐fold seasonal decrease in Whidbey and a twofold seasonal increase in South Sound (Fig. 8). CD in Central basin did not exhibit similar trends with other metrics, with its highest value in spring (2.1–2.6‰).

### 3.3 Environmental determinants of trophic structure

We found positive correlations between overall trophic diversity and the degree of terrestrial–marine connectivity as PC1 was positively correlated with community‐level SEA_c_ (*r *=* *.66, *p *<* *.01), CR (*r *=* *.55, *p *=* *.02), NR (*r *=* *.59, *p *=* *.01), and CD (*r *=* *.42, *p *=* *.08). Interestingly, at the assemblage level, fish niche widths and jellyfish niche widths showed contrasting associations with explanatory variables. Fish were positively correlated with PC1 (*r *=* *.63, *p *=* *.02), while jellyfish niche widths were not, and in contrast showed a negative trend with PC2 such that their niche widths decreased with urbanized land cover (*r = *−.40, *p *=* *.13). Correlations between predictor variables and metrics of trophic overlap were not significant (Table 3), although fish–jellyfish overlap showed a positive trend with PC1 (*r *=* *.3, *p *=* *.25) and a negative trend with PC3 (*r *=* *−.34, *p *=* *.19).

**Table 3:** Pearson correlations (*r*) between trophic structure metrics and the first three principal components

Metric	PC1		PC2		PC3	
	Flow (+), Shoreline Length (+), Salinity (−), FW‐NS Zooplankton (+)		% Developed (+), % Impervious (+), Gelatinous Zooplankton (+)		% Agriculture (−), Chl‐*a* (+), [Nitrate] (−), [Silicate] (−), Ichthyoplankton (+)	
	*r*	*p*‐Value	*r*	*p*‐Value	*r*	*p*‐Value
Assemblage						
Fish SEA_c_	**.63**	**.01**	.09	.72	−.11	.68
Jellyfish SEA_c_	.04	.89	−*.39*	*.13*	.03	.91
Jellyfish–fish Overlap	.04	.88	−.01	.96	−.04	.88
Fish–jellyfish Overlap	*.30*	*.25*	.09	.74	−*.34*	*.19*
Community						
SEA_c_	**.66**	**<.01**	−.14	.59	−.14	.57
CR	**.55**	**.02**	.10	.69	−.18	.47
NR	**.59**	**.01**	−.22	.37	−.09	.72
CD	*.41*	*.09*	.18	.48	−*.31*	*.21*

*p*‐Values denote the probability a given correlation coefficient is 0. Variables with significant loadings (>.3) on a given axis are listed in the heading along with the direction (+ or −). Significant correlations (*p *<* *.05) are shown in bold, and moderate (*r* > .3) correlations are shown in italics.

## 4 Discussion

### 4.1 General patterns at the species, assemblage, and community level

Our findings provide a broad picture of pelagic fish and jellyfish trophic structure across an urbanizing estuary. At minimum, this work advances the fundamental description of trophic ecology in these fish and jellyfish taxa, for which there is often limited information. In some cases, our results were unexpected. For instance, forage fish stickleback and surf smelt, which are generally considered planktivorous, often occupied the highest trophic levels where they occurred. While uncertainty in species‐specific trophic enrichment factors may have played a role, more detailed study into the trophic ecology of these taxa is clearly needed. Similarly, we were surprised by the weak effects of body size and the lack of evidence for consistent seasonal diet shifts for many of the species. While seasonal diet shifts associated with ontogeny have been documented for salmonids (Duffy, Beauchamp, Sweeting, Beamish, & Brennan, 2010) and some jellyfish (Fleming, Harrod, Newton, & Houghton, 2015), our sampling may not have captured these changes, especially if ontogenetic niche shifts were accompanied by changes in habitat use, for instance, movements to more offshore or deeper waters (Duffy et al., 2010; Moriarty, Andrews, Harvey, & Kawase, 2012).

The striking variability among basins and seasons we observed in fish–jellyfish trophic structure at the species, assemblage, and community level is consistent with previous studies in the region that noted spatiotemporal variability in community structure (Greene et al., 2015; Rice, Duda, Greene, & Karr, 2012) and abiotic conditions (Moore et al., 2008; Strickland, 1983). Consequently, a major implication from our study is the inference that trophic structure of pelagic fish and jellyfish is context dependent and locally controlled across a large heterogeneous landscape.

Fish–jellyfish trophic interactions also appeared to be highly context dependent. Overall, jellyfish were often more enriched in ^13^C and depleted in ^15^N than fish, possibly reflecting a more marine‐influenced diet. While low assemblage‐level overlap values corroborated this interpretation, there were important exceptions. For instance, assemblage‐level overlap still varied fourfold across basins and seasons and was notably high in South Sound in the spring. Moreover, there were numerous instances at the species level where error bars around fish and jellyfish substantially overlapped in biplot space. Similar studies in temperate waters have found variability in the extent of fish–jellyfish diet overlap to be strongly dependent on what species are being compared (Brodeur et al., 2002; Purcell & Sturdevant, 2001). Interestingly, this was not the case in our study as overlap between any pair of species appeared to vary unpredictably among basins and seasons, suggesting that the extent of fish–jellyfish trophic overlap in Puget Sound is locally controlled. However, we cannot infer specific factors given assemblage‐level ellipse overlap ratio was unrelated to the environmental gradients we measured. This may be due to aggregating two ecologically diverse groups (further discussed in *Caveats and Implications*), but we also speculate that abiotic and biotic attributes (e.g., prey composition, water clarity) at smaller spatial scales than our analysis captured may play a role and warrant future study.

### 4.2 Environmental drivers of assemblage‐ and community‐level trophic structure

Because our study is observational, we cannot fully resolve what caused the spatiotemporal patterns in trophic structure we observed. However, two lines of correlative evidence suggest that assemblage‐ and community‐level trophic structure was influenced by terrestrial connectivity. First, community‐level trophic diversity metrics (NR, CR, and SEA_c_) and niche widths of fish assemblages were significantly positively correlated with landscape gradients in connectivity; second, other biotic explanations, including variable species richness (Table S3), ontogenetic diet or habitat shifts (Fig. S1), and intrinsic trophic baseline variability (Table S2) appear insufficient to fully explain patterns.

The most plausible link between terrestrial connectivity and trophic structure is freshwater input, which drives heterogeneity in numerous biophysical conditions in estuaries, for example, productivity, water clarity, and organic matter dynamics (Kimmerer, 2002). In turn, this heterogeneity can influence trophic structure by increasing the diversity of distinct basal resources (Abrantes et al., 2013; Deegan & Garritt, 1997; Nelson, Deegan, & Garritt, 2015). For instance, inputs of terrestrial and river borne organic matter (Ruesink, Roegner, Dumbauld, Newton, & Armstrong, 2003) and benthic mixing (Simenstad & Wissmar, 1985) associated with watershed influence can increase coupling of terrestrial, littoral, and benthic carbon sources to pelagic food webs (Atwood, Wiegner, & MacKenzie, 2012; Martinetto, Teichberg, & Valiela, 2006). In turn, these pathways propagate through the food web resulting in greater trophic diversity of consumers. Alternatively, freshwater inputs can directly alter the prey base for fish and jellyfish by modifying zooplankton community structure and contributing subsidies of lotic and terrestrial invertebrates (Duffy et al., 2010; Romanuk & Levings, 2005).

The contrasting associations between fish and jellyfish with terrestrial connectivity may indicate direct effects on the prey base and suggests fish, especially salmonids, may be able to utilize a wider resource base in more terrestrially influenced habitats. However, assuming a 0.4‰ enrichment of ^13^C per trophic level (Post, 2002), the range of δ^13^C for nonsalmonid fish species (−20 to −15) relative to the range of terrestrial end members (−32 to −26) suggests direct incorporation was unlikely. Because we do not have complementary measures of fish and jellyfish diets or isotopes of lower trophic levels, we cannot determine the extent these patterns arose through differences in basal energy pathways vs. direct effects on the prey base.

In addition to being smaller, jellyfish niche width associations to environmental gradients were weaker than that of fish; however, they were moderately negatively correlated to urbanization. When coupled with other findings that jellyfish are more numerically abundant (Greene et al., 2015; Rice et al., 2012) and depleted in ^15^N in more urbanized sites (S. M. Naman, Unpublished), this may provide supportive evidence for jellyfish shifting to lower energy food chains with increasing urbanization, a mechanism proposed by some (e.g., Parsons & Lalli, 2002) but largely untested in the field. One interesting nuance from this interpretation is that jellyfish, despite being considered generalists (Richardson et al., 2009), may have more specialized diets (i.e., a smaller niche width) than fish in areas where they proliferate. This is consistent with previous suggestions that diet specialization by jellyfish on abundant lower trophic‐level prey is a key trait promoting mass aggregations (Dawson & Hamner, 2009; Wintzer, Meek, & Moyle, 2011). However, due to low sample size and lack of lower trophic‐level isotope measurements, this interpretation remains speculative.

### 4.3 Caveats and implications

There are numerous sources of uncertainty associated with stable isotopes (Layman et al., 2012), only some of which we could directly address. Trophic fractionation (TEF) is perhaps the most serious lingering source of uncertainty in our analysis, which incorporated a wide range of taxa. While we used the generally accepted value of 3.4‰, TEF can vary substantially among and within taxa (Vander Zanden, 2001). Consequently, trophic‐level designations and assemblage‐level comparisons should be interpreted cautiously. In addition, spatial, temporal, and taxonomic aggregation that was necessary to calculate community metrics complicates interpretation and may have reduced our power to detect patterns. For example, our analysis did not incorporate landscape variation *within* basins, which may influence trophic dynamics at finer scales, perhaps explaining why only weak correlations with urbanization were found. Similarly, aggregating fish and jellyfish into broad groupings clearly ignores substantial variation in trophic ecology among individual taxa, for example, between carnivorous and planktivorous jellyfish (Fleming et al., 2015; Nagata et al., 2015), and may have contributed to the ambiguity in the trophic overlap results.

While inferences from our results should be cautious, interpretable patterns were still strong enough to be detected; thus, the work still has several important implications. First, with respect to fish–jellyfish interactions, our study is among the first to examine the trophic ecology of these taxa seasonally across a large heterogeneous landscape. The divergent spatiotemporal patterns between fish and jellyfish niche widths is an important result in itself as it suggests that disparate factors may underpin the trophic ecology of these taxa. Similarly, variation in trophic positions among species and assemblage‐level overlap across the landscape indicates context dependency in their interactions. Future work should focus on better defining the specific conditions driving fish–jellyfish interactions and its connection to the relative abundances of these taxa.

Second, our study provides evidence that terrestrial connectivity, likely through freshwater influence, is a key factor influencing emergent assemblage and community‐level trophic diversity of pelagic fish and jellyfish. While the importance of connectivity is well documented for benthic and intertidal estuarine food webs (Choy, An, & Kang, 2008; Mcclelland, Valiela, & Michener, 2011), its importance to pelagic food webs has been less certain (Martinetto et al., 2006). Connectivity varies spatially across Puget Sound due to the configuration of large river deltas and the proximity of basins to the Fraser River, a substantial nonlocal source of freshwater (Banas et al., 2014). This spatial heterogeneity, coupled with contrasting seasonal patterns between snowmelt vs. rainfall‐dominated rivers, contributes to asynchronous freshwater influence among basins (Moore et al., 2008). Asynchrony is important for food web structure (e.g., McCann & Rooney, 2009), but empirical demonstrations of the extrinsic or intrinsic processes generating asynchrony in food webs are rare (Vasseur, Gaedke, & Mccann, 2005). One interpretation of our results could be that spatiotemporal asynchrony in hydrology among rivers across Puget Sound drives a dynamic mosaic of trophic structure and diversity across the landscape. In a management context, this suggests that alteration of terrestrial–marine connections may have wide ranging indirect impacts to pelagic food webs. In the face of projected increases in population density in Puget Sound and many other coastal areas, this calls for landscape‐level management incorporating these cross‐ecosystem linkages (Lindenmayer et al., 2008) and an increased focus on further defining the mechanistic pathways and overall extent of terrestrial influence on pelagic food webs.

More generally, our work highlights the utility of community‐level SIA to examine spatial and temporal variation in trophic structure across large heterogeneous landscapes. Given the shift toward more holistic ecosystem‐based management approaches (Harvey, Williams, & Levin, 2012; Rombouts et al., 2013), the development of empirical techniques to measure trophic structure in natural systems is critical (Wilson & Devlin, 2013). Caveats notwithstanding, community‐level stable isotope metrics provide a quantification of trophic structure that integrates numerous processes that are difficult to measure individually. For instance, despite substantial spatial and taxonomic aggregation, we were still able to relate assemblage‐ and community‐level trophic structure to environmental gradients. Still, the trophic ecology of coastal pelagic systems and the role of human influence as a determinant of pelagic food web shifts are still far from resolved. Future work may benefit from incorporation of smaller scale mechanistic approaches (e.g., mixing models) and experiments to complement the comparative landscape scale approach presented here.

## Funding Information

US EPA National Estuary Program. EPA DW‐13‐923‐278‐01

## Conflicts of Interest

None declared.

## Data Accessibility

Stable isotope data in Table S1. Other raw data available on request.
