# Supplementary material for: Stable isotope‐based trophic structure of pelagic fish and jellyfish across natural and anthropogenic landscape gradients in a fjord estuary
Source: Ecol Evol. 2016 Oct 18;6(22):8159–73. doi: 10.1002/ece3.2450 (PMC5108267; doi:10.1002/ece3.2450)
Supplement: Supplementary file 1 [file ECE3-6-8159-s001.docx]

**Supporting Information**

**Table S1:** Sample sizes (*n*), mean (± SD) trophic level (TL) and δ^13^C for each species in each basin and season. Trophic level calculations are described in *Materials and Methods* section δ^13^C values are corrected for lipid content for cases where C:N exceeded 3.5.

|  | **Species** | **Basin** | **Season** | ***n*** | **TL** | **± SD** | **δ^13^C** | **± SD** |
| --- | --- | --- | --- | --- | --- | --- | --- | --- |
| *Fish* |  |  |  |  |  |  |  |  |
|  | Chinook | Rosario | Spring | 33 | 2.85 | 0.22 | -17.76 | 1.29 |
|  |  |  | Summer | 15 | 2.69 | 0.56 | -19.19 | 2.98 |
|  |  |  | Fall | 6 | 2.14 | 0.54 | -20.19 | 1.13 |
|  |  | Whidbey | Spring | 24 | 2.53 | 0.72 | -20.81 | 3.83 |
|  |  |  | Summer | 43 | 2.63 | 0.84 | -19.84 | 2.91 |
|  |  |  | Fall | 7 | 2.95 | 0.76 | -19.73 | 3.66 |
|  |  | Admiralty | Spring | 3 | 2.88 | 0.19 | -17.99 | 0.03 |
|  |  |  | Summer | 18 | 3.25 | 0.25 | -17.19 | 1.27 |
|  |  |  | Fall | 1 | 2.94 | 0.00 | -19.03 | 0.00 |
|  |  | Hood Canal | Spring | 35 | 3.03 | 0.28 | -18.26 | 1.70 |
|  |  |  | Summer | 34 | 3.25 | 0.33 | -19.63 | 1.01 |
|  |  |  | Fall | 14 | 3.17 | 0.24 | -19.50 | 1.02 |
|  |  | Central | Spring | 32 | 3.15 | 0.32 | -19.53 | 1.86 |
|  |  |  | Summer | 27 | 2.95 | 0.54 | -17.71 | 2.52 |
|  |  |  | Fall | 13 | 2.93 | 0.55 | -18.03 | 4.61 |
|  |  | South Sound | Spring | 34 | 2.50 | 0.14 | -17.26 | 1.52 |
|  |  |  | Summer | 61 | 2.67 | 0.48 | -16.98 | 2.28 |
|  |  |  | Fall | 6 | 2.27 | 0.72 | -19.70 | 5.19 |
|  | Chum | Rosario | Spring | 22 | 2.78 | 0.32 | -16.85 | 2.01 |
|  |  |  | Summer | 1 | 3.32 | 0.00 | -17.01 | 0.00 |
|  |  | Whidbey | Spring | 6 | 3.04 | 0.12 | -17.65 | 2.35 |
|  |  |  | Summer | 10 | 2.88 | 0.65 | -18.72 | 4.01 |
|  |  | Admiralty | Spring | 13 | 3.00 | 0.13 | -17.45 | 3.55 |
|  |  |  | Summer | 6 | 3.09 | 0.11 | -17.16 | 1.25 |
|  |  |  | Fall | 1 | 3.38 | 0.00 | -16.05 | 0.00 |
|  |  | Hood Canal | Spring | 12 | 2.96 | 0.12 | -17.52 | 1.46 |
|  |  |  | Summer | 3 | 2.88 | 0.03 | -20.77 | 0.20 |
|  |  | Central | Spring | 7 | 3.10 | 0.18 | -17.44 | 1.29 |
|  |  |  | Summer | 27 | 2.92 | 0.24 | -15.89 | 0.76 |
|  |  |  | Fall | 1 | 3.18 | 0.00 | -17.61 | 0.00 |
|  |  | South Sound | Spring | 11 | 2.70 | 0.12 | -15.62 | 0.28 |
|  |  |  | Summer | 26 | 2.75 | 0.13 | -15.53 | 0.38 |
|  |  |  | Fall | 1 | 3.15 | 0.00 | -15.88 | 0.00 |
|  | Coho | Rosario | Spring | 1 | 1.58 | 0.00 | -26.56 | 0.00 |
|  |  | Whidbey | Spring | 4 | 2.52 | 0.86 | -21.18 | 2.53 |
|  |  |  | Summer | 2 | 3.25 | 0.13 | -18.98 | 0.51 |
|  |  | Hood Canal | Spring | 3 | 2.75 | 0.16 | -22.53 | 3.91 |
|  |  | Central | Spring | 2 | 3.17 | 0.09 | -18.48 | 0.42 |
|  |  |  | Summer | 3 | 2.80 | 0.16 | -18.62 | 2.84 |
|  |  | South Sound | Spring | 1 | 2.57 | 0.00 | -29.98 | 0.00 |
|  | Sockeye | Whidbey | Summer | 1 | 3.17 | 0.00 | -21.44 | 0.00 |
|  | Pacific herring | Rosario | Spring | 3 | 2.91 | 0.12 | -17.00 | 0.33 |
|  |  |  | Summer | 20 | 2.98 | 0.13 | -17.95 | 0.50 |
|  |  |  | Fall | 6 | 2.76 | 0.11 | -18.15 | 0.16 |
|  |  | Whidbey | Spring | 14 | 3.21 | 0.22 | -17.23 | 0.57 |
|  |  |  | Summer | 26 | 3.23 | 0.18 | -17.67 | 0.39 |
|  |  |  | Fall | 17 | 3.05 | 0.08 | -18.07 | 0.38 |
|  |  | Admiralty | Spring | 5 | 2.66 | 0.37 | -17.50 | 0.90 |
|  |  |  | Summer | 5 | 2.99 | 0.18 | -18.05 | 0.54 |
|  |  | Hood Canal | Spring | 2 | 3.00 | 0.11 | -18.28 | 0.95 |
|  |  |  | Summer | 3 | 2.91 | 0.16 | -17.81 | 0.18 |
|  |  |  | Fall | 9 | 3.01 | 0.18 | -19.57 | 1.18 |
|  |  | Central | Spring | 2 | 2.83 | 0.42 | -16.45 | 0.09 |
|  |  |  | Summer | 18 | 3.08 | 0.08 | -16.94 | 0.58 |
|  |  |  | Fall | 6 | 3.16 | 0.15 | -16.78 | 1.07 |
|  |  | South Sound | Spring | 1 | 2.35 | 0.00 | -16.07 | 0.00 |
|  |  |  | Summer | 8 | 2.88 | 0.13 | -15.19 | 0.78 |
|  |  |  | Fall | 1 | 2.58 | 0.00 | -18.36 | 0.00 |
|  | Surf smelt | Rosario | Spring | 2 | 3.07 | 0.14 | -16.53 | 0.64 |
|  |  |  | Summer | 16 | 3.07 | 0.15 | -17.54 | 0.44 |
|  |  |  | Fall | 1 | 2.85 | 0.00 | -16.60 | 0.00 |
|  |  | Whidbey | Spring | 14 | 3.08 | 0.18 | -17.77 | 0.98 |
|  |  |  | Summer | 37 | 3.13 | 0.13 | -18.16 | 0.87 |
|  |  |  | Fall | 15 | 3.17 | 0.21 | -17.93 | 0.59 |
|  |  | Admiralty | Spring | 1 | 2.76 | 0.00 | -16.78 | 0.00 |
|  |  |  | Summer | 9 | 2.97 | 0.10 | -16.32 | 0.33 |
|  |  | Hood Canal | Spring | 2 | 3.44 | 0.11 | -17.85 | 0.42 |
|  |  |  | Summer | 1 | 3.08 | 0.00 | -17.07 | 0.00 |
|  |  |  | Fall | 2 | 3.21 | 0.35 | -17.81 | 0.92 |
|  |  | Central | Summer | 1 | 3.17 | 0.00 | -18.16 | 0.00 |
|  |  |  | Fall | 8 | 3.26 | 0.09 | -16.45 | 0.35 |
|  |  | South Sound | Summer | 5 | 2.56 | 0.22 | -15.84 | 0.36 |
|  |  |  | Fall | 1 | 2.70 | 0.00 | -15.66 | 0.00 |
|  | Three-spined stickleback | Rosario | Spring | 9 | 3.01 | 0.15 | -17.95 | 0.44 |
|  |  |  | Summer | 5 | 3.07 | 0.08 | -17.86 | 0.42 |
|  |  |  | Fall | 2 | 2.97 | 0.14 | -17.10 | 0.12 |
|  |  | Whidbey | Spring | 10 | 3.46 | 0.17 | -17.90 | 0.91 |
|  |  |  | Summer | 12 | 3.32 | 0.12 | -17.77 | 0.57 |
|  |  |  | Fall | 4 | 3.40 | 0.11 | -17.78 | 0.49 |
|  |  | Admiralty | Spring | 1 | 3.61 | 0.00 | -19.42 | 0.00 |
|  |  |  | Summer | 2 | 3.42 | 0.05 | -17.09 | 1.26 |
|  |  | Hood Canal | Spring | 12 | 3.51 | 0.12 | -18.11 | 0.44 |
|  |  |  | Summer | 10 | 3.46 | 0.20 | -19.05 | 0.41 |
|  |  |  | Fall | 7 | 3.32 | 0.18 | -18.72 | 3.07 |
|  |  | Central | Spring | 1 | 3.37 | 0.00 | -18.45 | 0.00 |
|  |  |  | Summer | 6 | 3.37 | 0.17 | -17.72 | 0.91 |
|  |  |  | Fall | 2 | 3.48 | 0.19 | -17.22 | 1.01 |
|  |  | South Sound | Spring | 2 | 3.06 | 0.01 | -17.31 | 0.49 |
|  |  |  | Summer | 4 | 3.27 | 0.32 | -16.28 | 1.16 |
|  |  |  | Fall | 1 | 3.32 | 0.00 | -15.70 | 0.00 |
|  | Pacific sandlance | Rosario | Spring | 2 | 2.95 | 0.07 | -17.25 | 0.32 |
|  |  |  | Fall | 4 | 2.69 | 0.08 | -17.98 | 0.55 |
|  |  | Whidbey | Summer | 3 | 2.23 | 1.49 | -18.77 | 1.79 |
|  |  |  | Fall | 4 | 3.06 | 0.10 | -17.95 | 0.36 |
|  |  | Central | Spring | 1 | 2.75 | 0.00 | -18.41 | 0.00 |
|  |  |  | Summer | 1 | 3.02 | 0.00 | -17.12 | 0.00 |
|  |  | South Sound | Spring | 4 | 2.77 | 0.09 | -15.73 | 0.29 |
|  | Northern anchovy | Rosario | Spring | 1 | 3.60 | 0.00 | -16.71 | 0.00 |
|  |  |  | Summer | 2 | 3.05 | 0.36 | -18.22 | 1.16 |
|  |  | Whidbey | Spring | 3 | 3.58 | 0.16 | -17.09 | 1.52 |
|  |  |  | Summer | 2 | 3.44 | 0.09 | -17.18 | 0.15 |
|  |  |  | Fall | 1 | 2.74 | 0.00 | -18.38 | 0.00 |
|  |  | Hood Canal | Spring | 1 | 3.58 | 0.00 | -18.82 | 0.00 |
|  |  | Central | Fall | 1 | 2.86 | 0.00 | -17.39 | 0.00 |
|  | River lamprey | Rosario | Summer | 2 | 3.11 | 0.77 | -16.70 | 4.51 |
|  |  | Rosario | Fall | 1 | 3.11 | 0.00 | -15.12 | 0.00 |
|  |  | Whidbey | Spring | 3 | 1.77 | 1.00 | -17.02 | 2.44 |
|  |  | Whidbey | Summer | 3 | 1.83 | 0.64 | -19.84 | 3.02 |
|  | Pacific sand fish | Rosario | Summer | 1 | 3.38 | 0.00 | -16.67 | 0.00 |
|  | Bay pipefish |  | Summer | 1 | 2.99 | 0.00 | -16.31 | 0.00 |
|  |  |  | Fall | 2 | 3.03 | 0.15 | -13.27 | 2.59 |
|  |  | Whidbey | Spring | 2 | 3.16 | 0.29 | -11.96 | 0.12 |
|  |  |  | Summer | 1 | 3.78 | 0.00 | -14.38 | 0.00 |
|  |  |  | Fall | 1 | 3.15 | 0.00 | -11.91 | 0.00 |
|  |  | Hood Canal | Spring | 1 | 3.17 | 0.00 | -15.13 | 0.00 |
|  |  |  | Summer | 3 | 3.17 | 0.14 | -16.79 | 1.95 |
|  |  | Central | Summer | 2 | 2.73 | 0.61 | -14.37 | 0.48 |
|  |  |  | Fall | 1 | 3.21 | 0.00 | -13.63 | 0.00 |
|  |  | South Sound | Fall | 1 | 3.06 | 0.00 | -13.90 | 0.00 |
|  | Starry flounder | Rosario | Spring | 2 | 3.06 | 0.07 | -13.47 | 1.35 |
|  |  |  | Summer | 1 | 3.01 | 0.00 | -18.19 | 0.00 |
|  |  | Whidbey | Spring | 1 | 3.01 | 0.00 | -15.08 | 0.00 |
|  |  |  | Summer | 2 | 3.36 | 0.07 | -11.69 | 2.36 |
|  |  |  | Fall | 2 | 3.35 | 0.05 | -12.71 | 1.16 |
|  |  | Hood Canal | Fall | 1 | 2.76 | 0.00 | -15.25 | 0.00 |
|  |  | Central | Fall | 1 | 3.70 | 0.00 | -12.95 | 0.00 |
|  | Plainfin midshipman | Hood Canal | Spring | 1 | 3.48 | 0.00 | -19.00 | 0.00 |
|  | Tubesnout | Whidbey | Summer | 1 | 3.22 | 0.00 | -18.46 | 0.00 |
|  | Pacific tomcod | South Sound | Summer | 1 | 2.77 | 0.00 | -15.42 | 0.00 |
| *Jellyfish* |  |  |  |  |  |  |  |  |
|  | Sea gooseberry | Rosario | Spring | 7 | 2.63 | 0.09 | -17.31 | 0.64 |
|  |  |  | Summer | 15 | 2.68 | 0.26 | -17.64 | 1.32 |
|  |  |  | Fall | 10 | 2.76 | 0.09 | -17.14 | 0.57 |
|  |  | Whidbey | Spring | 5 | 2.81 | 0.16 | -17.30 | 0.52 |
|  |  |  | Summer | 3 | 2.96 | 0.21 | -17.44 | 0.76 |
|  |  |  | Fall | 1 | 3.10 | 0.00 | -17.69 | 0.00 |
|  |  | Admiralty | Summer | 5 | 2.57 | 0.13 | -15.80 | 0.88 |
|  |  |  | Fall | 5 | 2.61 | 0.00 | -17.43 | 0.00 |
|  |  | Hood Canal | Spring | 9 | 3.22 | 0.12 | -17.45 | 2.39 |
|  |  |  | Fall | 1 | 2.86 | 0.00 | -20.23 | 0.00 |
|  |  | Central | Spring | 9 | 2.66 | 0.35 | -16.28 | 0.41 |
|  |  |  | Summer | 6 | 2.91 | 0.16 | -15.93 | 0.90 |
|  |  | South Sound | Spring | 11 | 2.65 | 0.31 | -17.19 | 1.87 |
|  |  |  | Summer | 2 | 2.51 | 0.61 | -15.19 | 0.79 |
|  | Water jelly | Rosario | Spring | 4 | 2.46 | 0.17 | -16.13 | 0.34 |
|  |  |  | Summer | 2 | 2.45 | 0.29 | -15.68 | 0.56 |
|  |  |  | Fall | 1 | 2.86 | 0.00 | -16.68 | 0.00 |
|  |  | Whidbey | Spring | 3 | 2.79 | 0.11 | -14.48 | 0.79 |
|  |  |  | Summer | 13 | 2.88 | 0.23 | -16.21 | 1.33 |
|  |  | Admiralty | Spring | 3 | 2.72 | 0.20 | -15.85 | 0.44 |
|  |  |  | Summer | 9 | 2.62 | 0.25 | -14.57 | 0.64 |
|  |  |  | Fall | 1 | 2.40 | 0.00 | -15.14 | 0.00 |
|  |  | Hood Canal | Spring | 12 | 2.91 | 0.23 | -16.18 | 0.79 |
|  |  |  | Summer | 5 | 2.89 | 0.17 | -16.80 | 2.43 |
|  |  |  | Fall | 4 | 2.96 | 0.13 | -18.35 | 1.49 |
|  |  | Central | Spring | 13 | 2.59 | 0.18 | -15.25 | 0.85 |
|  |  |  | Summer | 20 | 2.78 | 0.20 | -14.97 | 0.66 |
|  |  |  | Fall | 2 | 3.06 | 0.04 | -13.75 | 0.20 |
|  |  | South Sound | Spring | 13 | 2.52 | 0.30 | -16.23 | 2.13 |
|  |  |  | Summer | 18 | 2.35 | 0.28 | -14.72 | 1.11 |
|  |  |  | Fall | 4 | 2.69 | 0.14 | -13.96 | 0.33 |
|  | Cross jelly | Rosario | Spring | 3 | 2.07 | 0.38 | -16.04 | 0.48 |
|  |  |  | Summer | 4 | 2.21 | 0.49 | -14.73 | 3.87 |
|  |  |  | Fall | 12 | 2.75 | 0.10 | -17.40 | 0.28 |
|  |  | Whidbey | Spring | 1 | 1.94 | 0.00 | -16.26 | 0.00 |
|  |  |  | Summer | 4 | 2.53 | 0.30 | -17.06 | 0.89 |
|  |  |  | Fall | 3 | 2.90 | 0.10 | -16.99 | 0.37 |
|  |  | Admiralty | Spring | 1 | 2.23 | 0.00 | -16.02 | 0.00 |
|  |  |  | Summer | 3 | 2.48 | 0.31 | -16.19 | 1.25 |
|  |  |  | Fall | 3 | 2.69 | 0.04 | -16.93 | 0.39 |
|  |  | Hood Canal | Spring | 4 | 2.32 | 0.19 | -16.23 | 0.43 |
|  |  |  | Fall | 1 | 2.68 | 0.00 | -16.17 | 0.00 |
|  |  | Central | Spring | 9 | 2.20 | 0.13 | -16.37 | 0.50 |
|  |  |  | Summer | 1 | 2.64 | 0.00 | -16.02 | 0.00 |
|  |  | South Sound | Spring | 7 | 2.16 | 0.22 | -15.98 | 0.46 |
|  | Lion’s mane | Rosario | Summer | 1 | 3.12 | 0.00 | -14.30 | 0.00 |
|  |  |  | Fall | 7 | 3.20 | 0.27 | -14.64 | 0.57 |
|  |  | Whidbey | Spring | 1 | 3.02 | 0.00 | -16.11 | 0.00 |
|  |  |  | Summer | 7 | 3.32 | 0.24 | -15.42 | 0.57 |
|  |  |  | Fall | 5 | 3.60 | 0.08 | -15.28 | 1.00 |
|  |  | Admiralty | Summer | 3 | 2.58 | 0.28 | -15.17 | 0.69 |
|  |  | Hood Canal | Spring | 10 | 2.92 | 0.30 | -16.32 | 0.77 |
|  |  |  | Summer | 8 | 3.12 | 0.33 | -17.09 | 1.41 |
|  |  |  | Fall | 5 | 3.27 | 0.14 | -15.72 | 0.59 |
|  |  | Central | Spring | 1 | 2.50 | 0.00 | -16.76 | 0.00 |
|  |  |  | Summer | 3 | 3.10 | 0.34 | -14.20 | 0.35 |
|  |  |  | Fall | 3 | 3.13 | 0.38 | -14.94 | 0.55 |
|  |  | South Sound | Summer | 13 | 2.55 | 0.51 | -14.77 | 0.87 |
|  |  |  | Fall | 6 | 3.03 | 0.12 | -14.25 | 0.60 |
|  | Moon jelly | Hood Canal | Spring | 2 | 2.27 | 0.10 | -19.89 | 0.35 |
|  |  |  | Summer | 5 | 2.59 | 0.31 | -20.15 | 0.43 |
|  |  | Central | Summer | 9 | 2.39 | 0.13 | -16.33 | 0.76 |
|  |  | South Sound | Spring | 1 | 2.79 | 0.00 | -15.61 | 0.00 |
|  |  |  | Summer | 6 | 2.10 | 0.23 | -16.68 | 0.34 |
|  |  |  | Fall | 2 | 2.40 | 0.28 | -16.37 | 0.63 |
|  |  | Central | Summer | 1 | 2.31 | 0.00 | -16.47 | 0.00 |
|  | Fried egg jelly | Whidbey | Summer | 2 | 2.92 | 0.26 | -16.50 | 0.40 |
|  |  | Hood Canal | Spring | 3 | 3.28 | 0.22 | -15.76 | 0.21 |
|  |  |  | Summer | 1 | 2.87 | 0.00 | -16.14 | 0.00 |
|  |  | Central | Summer | 5 | 3.15 | 0.25 | -14.15 | 0.52 |
|  |  | South Sound | Summer | 5 | 2.50 | 0.29 | -13.98 | 1.05 |
|  |  | South Sound | Fall | 3 | 3.72 | 0.00 | -13.58 | 0.00 |
|  | Clytia | Admiralty | Summer | 3 | 2.54 | 0.01 | -15.38 | 0.83 |
|  |  | Central | Summer | 3 | 2.66 | 0.29 | -15.58 | 0.25 |
|  |  | Hood Canal | Summer | 1 | 2.63 | 0.00 | -16.23 | 0.00 |
|  |  | Central | Summer | 2 | 2.80 | 0.05 | -16.08 | 0.59 |
|  |  | South Sound | Summer | 7 | 2.34 | 0.07 | -15.69 | 0.49 |
| *Cephalopods* | |  |  |  |  |  |  |  |
|  | Squid | Rosario | Spring | 2 | 2.93 | 0.06 | -16.78 | 0.06 |
|  |  | Whidbey | Fall | 1 | 3.51 | 0.00 | -16.85 | 0.00 |
|  |  | Admiralty | Summer | 1 | 2.89 | 0.00 | -16.32 | 0.00 |
|  |  | Central | Spring | 2 | 3.27 | 0.23 | -16.00 | 0.47 |
|  |  | South Sound | Spring | 3 | 2.85 | 0.24 | -15.32 | 0.37 |
|  |  |  | Summer | 1 | 3.24 | 0.00 | -14.08 | 0.00 |
|  |  |  | Fall | 1 | 2.84 | 0.00 | -15.51 | 0.00 |

**Appendix S1. Addressing Potential Caveats of Community-Level Isotope Analysis.**

*Trophic Baseline Variation*

There are several issues that may have confounded interpretation of our community-level analyses. First, it is possible that mid-trophic level patterns inferred from the isotope metrics may be obscured by spatiotemporal variation in the trophic baseline (e.g., Dethier et al. 2013). One drawback to the SIBER approach is that among-community differences in the variance of baseline end members can result in large differences in consumer-level metrics despite unchanged trophic structure (Hoeinghaus & Zeug 2008). Thus it is possible that landscape level patterns in trophic structure metrics in Puget Sound could be driven by systematic variation in the range of primary producer isotope values. We addressed this issue using a complementary set of particulate organic matter (POM) samples collected in June and September during our study. We complemented these data with POM data collected by Conway-Cranos *et al*. (2015), which used the same methodology, and provided a more rigorous temporal sampling of Rosario basin and Hood Canal.

While primary producer values did vary among basins, we did not find any systematic variation in the range between putative end members (Table S2). Consequently, baseline variation in producer δ^13^C is not a sufficient explanation for the patterns we observed. However, we recognize that the lack of complementary baseline measurements is a major limitation of our study.

**Table S2:** Mean δ^13^C (± SD) values of putative basal resource end members taken from other studies^*^ across Puget Sound. Values are aggregated across seasons.

| **Basin** | **Terrestrial δ^13^C** | **Macrophyte δ^13^C** |
| --- | --- | --- |
| Rosario | -30.6 (± 1.2) | -10.4 (± 2.5) |
| Whidbey | -26.5 (± 1.1) | -12.1 (± 1.0) |
| Admiralty | NA | NA |
| Hood Canal | -31.1 (± 2.1) | -13.2 (± 3.5) |
| Central | **-**32.5 **(**± 3.5) | -13.5 (± 4.1) |
| South Sound | **-**28.1 **(**± 0.4) | -13.1 (± 1.1) |

* References: Liedtke et al. 2011, Ruesink et al. 2014, Conway-Cranos et al. 2015, Howe and Simenstad 2015.

*Species Richness Effects*

Because species composition was not standardized, differences in trophic structure could be an artefact of spatial and seasonal differences in species composition and diversity. To address this possibility, we examined correlations between species richness and each trophic structure metric. Except for NR, which was positively correlated with richness (*r* = 0.64), we found no strong correlations (Table S3), suggesting our comparisons are robust to variable species numbers.

**Table S3:** Pearson Correlations between SIBER metrics and the number of species sampled in each basin-season combination.

*Habitat Shifts*

If consumers accrue a significant proportion of their tissue in a different habitat then where they were sampled, inferences into niche diversity may be confounded with ontogenetic habitat shifts. For instance, juvenile salmonids may accrue significant tissue in freshwater habitats before migration into the Puget Sound estuary (Duffy, Beauchamp & Buckley 2005), complicating our interpretation of C diversity among basins and seasons. We suspect this was not a significant issue in our study as the dominant salmonids (Chinook and chum) both generally exhibit ocean type life histories, where they migrate directly to saltwater after emergence (Quinn 2005). However, some Chinook do exhibit stream type life histories, such that depleted C values may reflect tissue accrued in freshwater habitats. To address this issue, we analyzed δ^13^C in the blood for a random subset (*n* = 30) of Chinook captured across all basins in April, May and June. Because blood has a faster turnover time than muscle tissue (Thomas & Crowther 2015), incongruent δ^13^C values between these tissue types within individuals (e.g., lower values in muscle relative to blood) could indicate habitat shifts. We found good correspondence between tissue types (Figure S1), suggesting these fish likely accrued their tissue in the estuary where they were sampled.

Habitat shifts may also be an issue for juvenile demersal fish, which may integrate benthic C pathways. While we are unable to rule out this possibility, these taxa comprised a small proportion of the community where they occurred, thus would only minimally influence SIBER metrics.


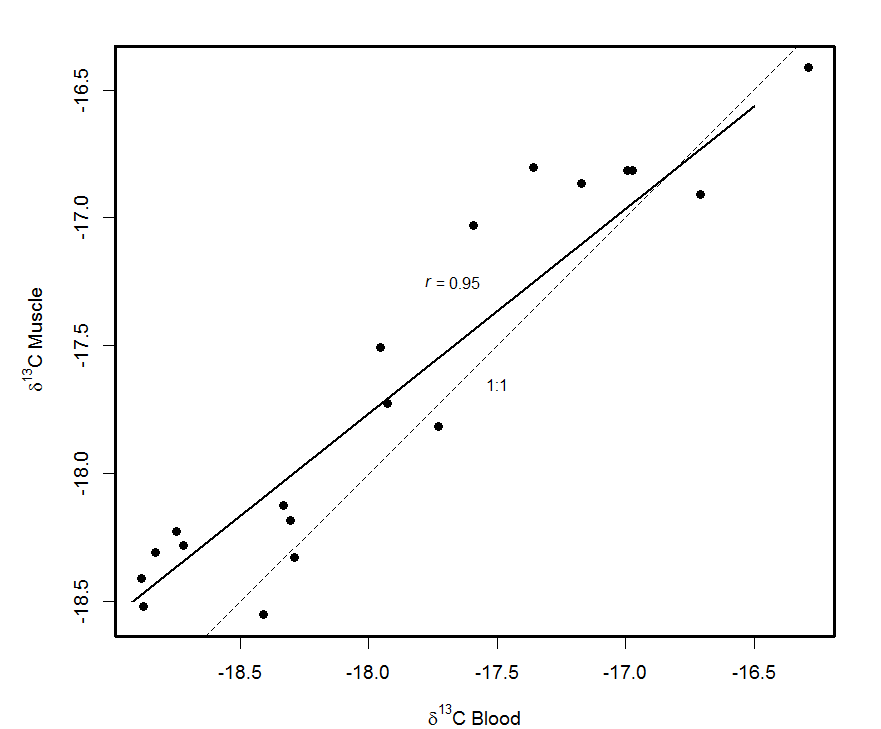


**Figure S1:** Relationship between δ^13^C in blood and muscle tissue from Chinook salmon collected in June. The linear trend is plotted as the solid line with the dashed 1:1 line as a reference.

**Appendix S2. The Effects of Body Size**

Variation in body size among individuals is well known to influence stable isotope values, especially δ^15^N (e.g. Vander Zanden 2001; Nagata *et al.* 2015). However, the strength of this relationship varies considerably and should strongly depend on species-specific life history characteristics and the range of sizes sampled (Jennings *et al.* 2001). For instance, δ^15^N-body size relationships should be stronger when sampling a single cohort through ontogenetic diet shifts as opposed to intra-individual variation across multiple cohorts. Our sampling design likely falls into the latter category for many fish species as all samples were taken from a single life stage. In contrast, multiple life stages were likely sampled for some jellyfish species (most notably fried egg and lion’s mane). Still given that the average body size of most species increased seasonally and varied among basins (C. Greene *Unpublished data*), we further explored how size variation influenced δ^15^N.

Simple linear regressions were performed relating body size, measured as fish fork length and jellyfish bell diameter, to δ^15^N and δ^13^C. For pooled jellyfish samples, the average bell diameter was used. A subset of species were selected for this analysis based on abundance (i.e. if they occurred in more than 20% of tows) and if there was a strong *a priori* expectation that multiple life stages were sampled. Assumptions of normality and homogeneity were evaluated by graphically examining residual plots and a log+1 transformation was used when cases deviated from these assumptions.

Body size was a significant predictor of δ^15^N (Table S4) for all but three taxa (water jelly, lion’s mane, and moon jelly). In all cases δ^15^N increased with body size. However, the proportion of variation explained (R^2^) was low and only exceeded 30% for two species (surf smelt and fried egg jelly). For δ^13^C, body size was a significant predictor only for Chinook and Pacific herring (Table S5) but explained less than 15% in each of these cases.

Taken together, results from this analysis indicate that while variation in body mass likely contributed to variation in aggregate trophic structure and the relative niche positions of individual species, its effect was weak relative to other factors. In addition, these analyses suggest that ontogenetic niche shifts, which should have been evident from strong size- δ^15^N relationships, either did not occur during our study period (fried egg jellyfish and surf smelt are possible exceptions) or our sampling design was not able to detect them.

**Table S4.** Results of linear regressions relating body length of fish and bell diameter of jellyfish to δ^15^N. The size range is also shown for each taxa.

|  |  |  |  |  |  |  | **Size Range (mm)** | | |  |
| --- | --- | --- | --- | --- | --- | --- | --- | --- | --- | --- |
|  | **Term** | **df** | **SS** | ***F*** | ***P-value*** | **R2** | **Min** | **Mean** | **Max** |  |
| Chinook, *Oncorhynchus tshawytscha* | |  |  |  |  |  |  |  |  |  |
|  | Length | 1 | 81.32 | 24.38 | 0 | 0.05 | 65 | 118 | 189 |  |
|  | Residuals | 435 | 1450.81 |  |  |  |  |  |  |  |
| Stickleback, *Gasterosteus aculeatus* | |  |  |  |  |  |  |  |  |  |
|  | Length | 1 | 5.25 | 12.41 | 0 | 0.12 | 34 | 67 | 91 |  |
|  | Residuals | 94 | 39.78 |  |  |  |  |  |  |  |
| Chum*, O. keta* | |  |  |  |  |  |  |  |  |  |
|  | Length | 1 | 16.23 | 24.42 | 0 | 0.16 | 79 | 123 | 171 |  |
|  | Residuals | 127 | 84.41 |  |  |  |  |  |  |  |
| Pacific herring*, Clupea pallasii* | |  |  |  |  |  |  |  |  |  |
|  | Length | 1 | 14.36 | 55.29 | 0 | 0.26 | 63 | 108 | 182 |  |
|  | Residuals | 156 | 40.51 |  |  |  |  |  |  |  |
| Surf smelt*, Hypomesus pretiosus* | |  |  |  |  |  |  |  |  |  |
|  | Length | 1 | 14.45 | 72.09 | 0 | 0.35 | 79 | 114 | 177 |  |
|  | Residuals | 132 | 26.45 |  |  |  |  |  |  |  |
| Water jelly*, Aequorea* spp. | |  |  |  |  |  |  |  |  |  |
|  | Diameter | 1 | 0.84 | 1.24 | 0.27 | 0.01 | 27 | 44 | 59 |  |
|  | Residuals | 90 | 60.98 |  |  |  |  |  |  |  |
| Lion's mane*, Cyanea capillata* | |  |  |  |  |  |  |  |  |  |
|  | Diameter | 1 | 0.79 | 0.45 | 0.5 | 0.01 | 21 | 241 | 410 |  |
|  | Residuals | 56 | 97.84 |  |  |  |  |  |  |  |
| Cross jelly*, Mitrocoma* spp. | |  |  |  |  |  |  |  |  |  |
|  | Diameter | 1 | 12.15 | 8.72 | 0.01 | 0.21 | 17 | 46 | 68 |  |
|  | Residuals | 33 | 45.97 |  |  |  |  |  |  |  |
| Fried egg *jelly, Phacellophora camtschatica* | |  |  |  |  |  |  |  |  |  |
|  | Diameter | 1 | 9.7 | 8.24 | 0.01 | 0.37 | 84 | 207 | 400 |  |
|  | Residuals | 14 | 16.47 |  |  |  |  |  |  |  |
| Moon jelly*, Aurelia* spp. | |  |  |  |  |  |  |  |  |  |
|  | Diameter | 1 | 0.36 | 0.53 | 0.48 | 0.03 | 55 | 91 | 152 |  |
|  | Residuals | 18 | 12.37 |  |  |  |  |  |  |  |

**Table S5.** Results of linear regressions relating body length of fish and bell diameter of jellyfish to δ^13^C. Size ranges are the same as Table S4.

|  | **Term** | **df** | **SS** | ***F*** | ***P*-value** | **R^2^** |
| --- | --- | --- | --- | --- | --- | --- |
| Chinook, *Oncorhynchus tshawytscha* | |  |  |  |  |  |
|  | Length | 1 | 132.31 | 17.68 | <0.01 | 0.04 |
|  | Residuals | 394 | 2948.39 |  |  |  |
| Stickleback, *Gasterosteus aculeatus* | |  |  |  |  |  |
|  | Length | 1 | 3.36 | 3.51 | 0.06 | 0.04 |
|  | Residuals | 77 | 73.78 |  |  |  |
| Chum*, O. keta* | |  |  |  |  |  |
|  | Length | 1 | 1.89 | 0.44 | 0.51 | 0.00 |
|  | Residuals | 112 | 476.43 |  |  |  |
| Pacific herring*, Clupea pallasii* | |  |  |  |  |  |
|  | Length | 1 | 13.33 | 14.73 | <0.01 | 0.10 |
|  | Residuals | 135 | 122.15 |  |  |  |
| Surf smelt*, Hypomesus pretiosus* | |  |  |  |  |  |
|  | Length | 1 | 0.82 | 0.98 | 0.33 | 0.01 |
|  | Residuals | 109 | 91.76 |  |  |  |
| Water jelly*, Aequorea* spp. | |  |  |  |  |  |
|  | Diameter | 1 | 2.03 | 0.89 | 0.35 | 0.01 |
|  | Residuals | 90 | 204.80 |  |  |  |
| Lion's mane*, Cyanea capillata* | |  |  |  |  |  |
|  | Diameter | 1 | 65.93 | 5.05 | 0.03 | 0.08 |
|  | Residuals | 56 | 731.18 |  |  |  |
| Cross jelly*, Mitrocoma* spp. | |  |  |  |  |  |
|  | Diameter | 1 | 8.58 | 5.45 | 0.03 | 0.14 |
|  | Residuals | 33 | 51.96 |  |  |  |
| Fried egg jelly*, Phacellophora camtschatica* | |  |  |  |  |  |
|  | Diameter | 1 | 0.02 | 0.02 | 0.88 | 0.00 |
|  | Residuals | 14 | 13.86 |  |  |  |
| Moon jelly*, Aurelia* spp. | |  |  |  |  |  |
|  | Diameter | 1 | 3.12 | 1.01 | 0.33 | 0.05 |
|  | Residuals | 18 | 55.53 |  |  |  |

**Appendix S3. Additional Zooplankton Collection Methodology**

To characterize community abundance and composition at lower trophic levels, we collected a horizontal surface plankton tow at each site. Following each fish trawl, a 1.0 m wide, 3.0 m long net with 500 µm mesh was towed for 3 minutes at a speed of 2 metric knots through the water in an arc to avoid disturbance from the boat’s wake. A General Oceanics^®^ flowmeter was attached at the center of the net to quantify the volume of water filtered. After the tow, net contents were elutriated through a 500 µm sieve and preserved in 10% neutral buffered formalin. All zooplankton were identified to the lowest possible taxonomic level in the laboratory using a dissecting microscope. For the purpose of our study, specimens were categorized into coarse groups based on taxonomy, functional feeding mode and habitat use (J. Cordell *Unpublished*). Specific groupings used in our analysis included: abundance of total zooplankton, icthyoplankton and gelatinous zooplankton; relative abundance of omnivore, predator and grazer feeding groups; and relative abundance of taxa associated with nearshore and freshwater habitats.

**References**

Conway-Cranos, L., Kiffney, P., Banas, N., Plummer, M., Naman, S., MacCready, P., Bucci, J. & Ruckelshaus, M. (2015) Stable isotopes and oceanographic modeling reveal spatial and trophic connectivity among terrestrial, estuarine, and marine environments. *Marine Ecology Progress Series*, **533**, 15–28.

Dethier, M., Sosik, E., Galloway, A., Duggins, D. & Simenstad, C. (2013) Addressing assumptions: variation in stable isotopes and fatty acids of marine macrophytes can confound conclusions of food web studies. *Marine Ecology Progress Series*, **478**, 1–14.

Duffy, E.J., Beauchamp, D.A. & Buckley, R.M. (2005) Early marine life history of juvenile Pacific salmon in two regions of Puget Sound. *Estuarine, Coastal and Shelf Science*, **64**, 94–107.

Hoeinghaus, D.J. & Zeug, S.C. (2008) Can stable isotope ratios provide for community-wide measures of trophic structure? Comment. *Ecology*, **89**, 2353–7; discussion 2358–9.

Howe, E. & Simenstad, C. (2015) Using stable isotopes to discern mechanisms of connectivity in estuarine detritus-based food webs. *Marine Ecology Progress Series*, **518**, 13–29.

Jennings, S., Pinnegar, J.K., Polunin, N.V.C. & Boon, T.W. (2001) Weak cross-species relationships between body size and trophic level belie powerful size-based trophic structuring in fish communities. *Journal of Animal Ecology*, **70**, 934–944.

Liedtke, T., Smith, C. & Rondorf, D. (2011) Stable isotopes of nitrogen and carbon as tools to monitor eutrophication and trophic dynamics. *Hydrography of and biochemical Inputs to Liberty Bay, a small urban embayment in Puget Sound, Washington* (ed R.K. Takesue), pp. 69–84. US. Geological Survey Scientific Investigations Report 2011-5152.

Nagata, R., Moreira, M., Pimentel, C. & Morandini, A. (2015) Food web characterization based on δ15N and δ13C reveals isotopic niche partitioning between fish and jellyfish in a relatively pristine ecosystem. *Marine Ecology Progress Series*, **519**, 13–27.

Quinn, T.P. (2005) *Behavior and Ecology of Pacific Salmon and Trout*. University of Washington Press.

Ruesink, J.L., Trimble, A.C., Berry, H., Sprenger, A.G. & Dethier, M.N. (2014) Environmental correlates of growth and stable isotopes in intertidal species along an estuarine fjord. *Estuaries and Coasts*, **37**, 149–159.

Thomas, S.M. & Crowther, T.W. (2015) Predicting rates of isotopic turnover across the animal kingdom: a synthesis of existing data. *Journal of Animal Ecology*, **84**, 861–870.

Vander Zanden, M.J. (2001) Variation in ␦ 15 N and ␦ 13 C trophic fractionation: implications for aquatic food web studies. *Limnology and Oceanography*, **46**, 2061–2066.
